# EBV-positive diffuse large B-cell lymphoma with central nervous system involvement in Anti-Mi-2-positive dermatomyositis

**DOI:** 10.1093/rap/rkag041

**Published:** 2026-03-28

**Authors:** Shion Kachi, Ran Nakashima, Hiroaki Ito, Ran Fujimoto, Tsuneo Sasai, Mirei Shirakashi, Ryosuke Hiwa, Hideaki Tsuji, Shuji Akizuki, Akira Onishi, Hajime Yoshifuji, Akio Morinobu

**Affiliations:** Department of Rheumatology and Clinical Immunology, Graduate School of Medicine, Kyoto University, Kyoto, Japan; Department of Rheumatology and Clinical Immunology, Graduate School of Medicine, Kyoto University, Kyoto, Japan; Department of Diagnostic Pathology, Kyoto University Hospital, Kyoto, Japan; Department of General Internal Medicine, Kobe City Medical Center General Hospital, Kobe, Japan; Department of Rheumatology and Clinical Immunology, Graduate School of Medicine, Kyoto University, Kyoto, Japan; Department of Rheumatology and Clinical Immunology, Graduate School of Medicine, Kyoto University, Kyoto, Japan; Department of Rheumatology and Clinical Immunology, Graduate School of Medicine, Kyoto University, Kyoto, Japan; Department of Rheumatology and Clinical Immunology, Graduate School of Medicine, Kyoto University, Kyoto, Japan; Department of Rheumatology and Clinical Immunology, Graduate School of Medicine, Kyoto University, Kyoto, Japan; Department of Advanced Medicine for Rheumatic Diseases, Graduate School of Medicine, Kyoto University, Kyoto, Japan; Department of Rheumatology and Clinical Immunology, Graduate School of Medicine, Kyoto University, Kyoto, Japan; Department of Rheumatology and Clinical Immunology, Graduate School of Medicine, Kyoto University, Kyoto, Japan

Key message

- Unexplained fever and confusion in methotrexate/tacrolimus-treated patients warrant clinical suspicion for Epstein-Barr virus-driven central nervous system immune deficiency/dysregulation lymphoproliferative disorders.

Dear Editor, Immune deficiency/dysregulation-associated lymphoproliferative disorders (IDD-LPDs) can occur during immunosuppressive therapy, including methotrexate (MTX) [1]. Although extranodal involvement is common, central nervous system (CNS) involvement is rare [1].

We report a 79-year-old woman with anti-Mi-2 antibody-positive dermatomyositis who developed Epstein–Barr virus (EBV)-related IDD-LPD with CNS involvement during treatment with MTX, prednisolone (PSL) and tacrolimus (TAC).

A 79-year-old Japanese woman with anti-Mi-2 antibody-positive dermatomyositis was admitted with fever, anorexia and fatigue. She had been diagnosed with dermatomyositis at the age of 75 and initially received remission induction therapy with methylprednisolone pulse therapy followed by high-dose PSL (1 mg/kg/day), MTX, TAC and intravenous immunoglobulin (IVIG). At the age of 76, she relapsed and was retreated with IVIG; MTX and TAC were increased to 12 mg/week and 6 mg/day, respectively. Thereafter, her disease activity remained controlled, and PSL was tapered to 3.5 mg/day.

Five days before admission, she developed a fever >38°C with anorexia and malaise. On admission, she was alert; temperature 36.4°C, blood pressure 106/72 mmHg, and SpO_2_ 96% on room air. Laboratory findings showed a white blood cell count of 4180/µl (atypical lymphocytes 7%), C-reactive protein (CRP) 26.2 mg/l, and soluble IL-2 receptor (sIL-2R) 1140 U/ml. Urinalysis showed pyuria and bacteriuria, and urine culture grew *Escherichia coli*, suspected with urinary tract infection. Immunosuppressants (MTX and TAC) were discontinued, and ceftriaxone was initiated.

On hospital day 2, she developed fever and impaired consciousness (Glasgow Coma Scale, 14). Given persistent fever and the development of nuchal rigidity, cerebrospinal fluid (CSF) examination was performed on day 4, demonstrating pleocytosis (55/µl, mononuclear predominance). The patient exhibited elevated protein levels (134.5 mg/dl) and relatively low glucose levels (76 mg/dl; serum 157 mg/dl). No significant organisms were isolated from the CSF culture. Magnetic resonance imaging revealed dural enhancement on the contrast-enhanced T1-weighted image, as well as high signal intensities along the cerebral sulci on the contrast-enhanced fluid-attenuated inversion recovery image (Fig. 1A). Despite broadened antimicrobial therapy and empiric antituberculosis therapy for suspected tuberculous meningitis, the patient’s fever and impaired consciousness continued.

**Figure 1:**
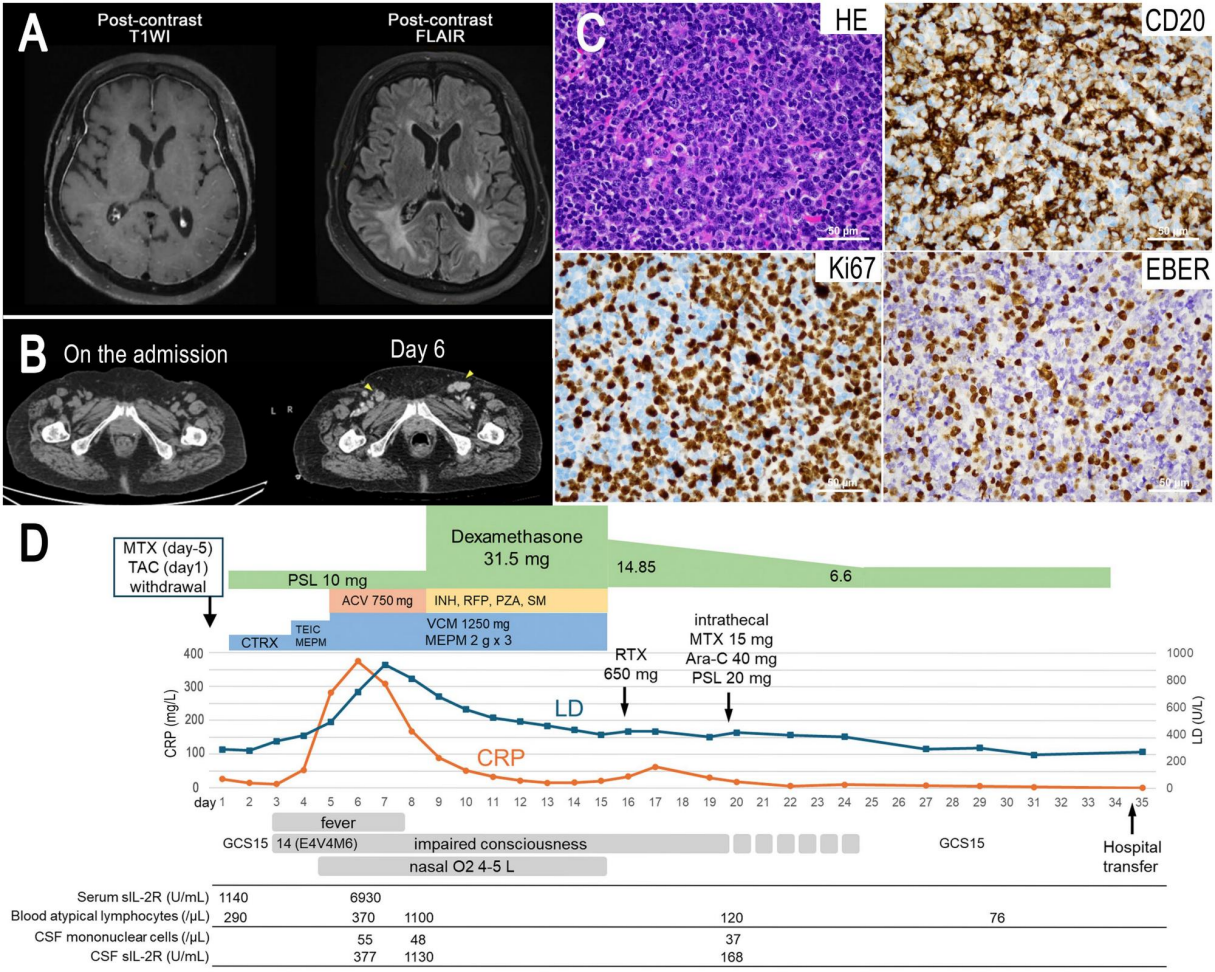
Imaging and clinical timeline. (A) The contrast-enhanced T1-weighted image (T1WI) on the left shows dural enhancement, and the contrast-enhanced fluid-attenuated inversion recovery (FLAIR) image on the right exhibits high signal intensities along the cerebral sulci. (B) Computed tomography (CT) images on admission and 6 days after admission. Yellow arrows indicate enlarged inguinal lymph nodes. (C) The inguinal lymph node biopsy showed a background of small lymphocytes with diffuse proliferation of large lymphocytes, consistent with EBER-positive diffuse large B-cell lymphoma (DLBCL). (D) Clinical timeline. Abbreviations: methotrexate (MTX); tacrolimus (TAC); teicoplanin (TEIC); meropenem (MEPM); vancomycin (VCM); acyclovir (ACV); isoniazid (INH); rifampicin (RFP); pyrazinamide (PZA); streptomycin (SM); rituximab (RTX); cytarabine (Ara-C); Glasgow Coma Scale (GCS); cerebrospinal fluid (CSF); soluble IL-2 receptor (sIL-2R)

On day 6, cervical and inguinal lymphadenopathy became apparent, with marked elevation of CRP 385 mg/l and sIL-2R 6930 U/ml. Contrast-enhanced CT (Fig. 1B) revealed newly developed cervical, axillary and inguinal lymphadenopathy and bilateral adrenal enlargement. Serology suggested prior EBV infection (EBV-VCA IgG positive, IgM negative; EBNA positive), and EBV DNA in blood was elevated (2700 copies/µg DNA). Inguinal lymph node biopsy revealed diffuse proliferation of large atypical lymphoid cells. Immunohistochemically, the tumor cells were positive for CD19, CD20, PAX5, CD79a, BCL6 and MUM1, and negative for CD3, CD5, and CD10. In situ hybridization for Epstein–Barr virus–encoded RNA (EBER) was positive. The Ki-67 labelling index was approximately 70% (Fig. 1C). These findings supported EBV-positive diffuse large B cell lymphoma (DLBCL), non-GCB type. The CSF cytology demonstrated atypical lymphocytes, and EBV DNA was detectable in stored CSF (90 copies/µg DNA), indicating CNS involvement. Given the preceding immunosuppressive therapy, including MTX, she was diagnosed with IDD-LPD.

After exclusion of alternative infectious etiologies, PSL was tapered and rituximab (500 mg/m^2^) was administered. Intrathecal chemotherapy (MTX 15 mg, cytarabine 40 mg and PSL 20 mg) was performed on day 20 (Fig. 1D). Over the subsequent 2 weeks, serum CRP levels, CSF mononuclear cell counts and CSF sIL-2R levels markedly decreased, and her consciousness fully recovered.

This case illustrates IDD-LPD with CNS involvement arising in dermatomyositis during combination immunosuppression with MTX, PSL and TAC. IDD-LPDs frequently present as extra nodal DLBCL [1], whereas cases occurring in dermatomyositis are limited [2]. Although evidence is mainly derived from RA cohorts [3], MTX dose has been associated with MTX-LPD risk, and doses ≥8 mg/week have been reported as a risk factor [4]. In this case, MTX was increased to 12 mg/week following a flare, which may have contributed to the risk.

EBV is implicated in LPD pathogenesis under immunosuppression. MTX may promote EBV-driven lymphomagenesis through impaired T cell surveillance and potential effects on viral activation pathways, although the *in vivo* relationship between MTX exposure, EBV load and anti-EBV immunity remains debated. Prior reports in idiopathic inflammatory myopathies describe EBV-related IDD-LPDs arising during immunosuppressants other than MTX [5], which often improve after discontinuation of immunosuppressive agents.

A 2023 systematic review of CNS IDD-LPDs reported that most cases were EBER-positive and much improved after MTX withdrawal, with additional treatments such as rituximab, chemotherapy or surgery, when necessary [6]. Early discontinuation of immunosuppressants is therefore central to management; if regression does not occur, glucocorticoids and/or lymphoma-directed therapy should be considered. The clinical significance of reintroducing MTX after remission remains uncertain.

This case highlights that EBV-positive CNS IDD-LPDs may complicate immunosuppressive therapy for anti–Mi-2 antibody-positive dermatomyositis. IDD-LPDs should be included in the differential diagnosis in older immunosuppressed patients, and timely withdrawal of immunosuppressive agents should be considered when suspected. (791/800 words).

## Data Availability

The data underlying this article will be shared on reasonable request to the corresponding author (R.N.).
